# Drivers of Vaginal Drug Delivery System Acceptability from Internet-Based Conjoint Analysis

**DOI:** 10.1371/journal.pone.0150896

**Published:** 2016-03-21

**Authors:** Rachel J. Primrose, Toral Zaveri, Alyssa J. Bakke, Gregory R. Ziegler, Howard R. Moskowitz, John E. Hayes

**Affiliations:** 1 Sensory Evaluation Center, The Pennsylvania State University, University Park, Pennsylvania, United States of America; 2 Department of Food Science, College of Agricultural Sciences, The Pennsylvania State University, University Park, Pennsylvania, United States of America; 3 Mind Genomics Associates Inc., White Plains, New York, United States of America; University of Pittsburgh, UNITED STATES

## Abstract

Vaginal microbicides potentially empower women to protect themselves from HIV and other sexually transmitted infections (STIs), especially when culture, religion, or social status may prevent them from negotiating condom use. The open literature contains minimal information on factors that drive user acceptability of women’s health products or vaginal drug delivery systems. By understanding what women find to be most important with regard to sensory properties and product functionality, developers can iteratively formulate a more desirable product. Conjoint analysis is a technique widely used in market research to determine what combination of elements influence a consumer’s willingness to try or use a product. We applied conjoint analysis here to better understand what sexually-active woman want in a microbicide, toward our goal of formulating a product that is highly acceptable to women. Both sensory and non-sensory attributes were tested, including shape, color, wait time, partner awareness, messiness/leakage, duration of protection, and functionality. Heterosexually active women between 18 and 35 years of age in the United States (n = 302) completed an anonymous online conjoint survey using IdeaMap software. Attributes (product elements) were systematically presented in various combinations; women rated these combinations of a 9-point willingness-to-try scale. By coupling systematic combinations and regression modeling, we can estimate the unique appeal of each element. In this population, a multifunctional product (i.e., broad spectrum STI protection, coupled with conception) is far more desirable than a microbicide targeted solely for HIV protection; we also found partner awareness and leakage are potentially strong barriers to use.

## Introduction

Human Immunodeficiency Virus (HIV) and other sexually transmitted infections (STIs) remain a global threat to public health. Around the world, nearly 36 million adults and children are estimated to live with HIV, and estimates suggest nearly 350 million adults are infected with other curable STIs [1, 2]. Despite sustained efforts to reduce HIV transmission via sexual activity and drug use, infections continue to increase. According to recent United Nations data, between 1.9 million and 2.2 million individuals were newly infected with HIV in 2014. In the United States, the Centers for Disease Control and Prevention (CDC) estimates there are 1.2 million individuals aged 13 and older living with HIV, with approximately 50,000 new infections each year [3]. In the United States, one in four individuals infected with HIV are women, and for women most infections occur through heterosexual contact [4]. According to the most recent state profile from CDC, Pennsylvania ranked 10^th^ out of 50 states for HIV incidence, with an estimated 1,419 new diagnoses in adults and adolescents in 2013 [5]. Of these, 38.1% occurred via heterosexual transmission [5]. Vaginal microbicides have the potential to significantly reduce the amount of new HIV and STI diagnoses in women and slow heterosexual transmission to men.

Vaginal microbicides are products that can protect against HIV exposure during coitus; they may contain tenofovir, dapivirine, or other antiretroviral drugs as active ingredients and come in various forms such as gels, films, tablets, and rings [6–10]. In other studies of vaginal microbicides, they have been well liked by women from diverse backgrounds and locations [11–15]. Although some clinical trials have shown encouraging results [16–18], no commercially available microbicides have reached the market to date, due to various factors which have negatively influenced their effectiveness in clinical trials [19–21]. Consumer acceptability has repeatedly been shown to influence user adherence and willingness to try the product, which in turn affects the product’s real world effectiveness [17, 20, 21]. Other reasons for poor adherence that have been identified include practical reasons (i.e., missed visits, lack of product replenishments, scheduling conflicts, forgetfulness, etc), social consequences (including stigma, and discrimination), partner complaints, knowledge of or beliefs about other participants non-use, side effects, fear of harm, and mistrust of stated research goals [21]. Women were also ambivalent about using powerful drugs when they had no illness [21]. A lack of demonstrated benefit was an important factor in non-use, given these various barriers to adherence [21]. Critically however, the CAPRISA004 trial found higher rates of adherence were associated with lower rates of HIV incidence, demonstrating that vaginal microbicides can be effective when adherence is high [17]. Thus, to maximize protocol compliance and product efficacy, it is critical that the design process results in a product that is both biologically efficacious and well accepted by the end user. This requires that formulators recognize potential barriers to adherence and devise delivery systems that avoid or ameliorate these effects.

Clearly, i*n vivo* assessment by women is the ultimate test of product acceptability and adherence. However, clinical trials are extremely expensive, time consuming, and personally invasive, which limits the number of product alternatives that can be explored. One way to overcome this is to first screen a wide range of diverse product concepts, and narrow the field of products that move forward in the development pipeline. Women are unlikely to try (or continue to use) products that they reject based on visual appearance or product concept. A set of potential prototypes can be further winnowed via *in mano* sensory evaluation (i.e., outside the body, in the hand), to further reduce the set of prototypes that need to be subsequently evaluated *in vivo*.

Vaginal microbicide researchers are paying increasing attention to user perception and sensory aspects of products, and how they relate to each other and to acceptance. Recently, Morrow and colleagues explored meaning-making in relation to sensory properties of vaginal products [22]. They found women express beliefs about products and efficacy based on *in mano* sensory evaluation, and the authors hypothesize these meanings affect women’s willingness-to-try and actual use of vaginal products. Similarly, prior work by our team suggests shape, size, and firmness all affected women’s reported willingness-to-try a hypothetical microbicide [13]. Moreover, we found the optimally acceptable size and firmness varied depending on whether or not an applicator would be provided with the microbicide [13].

Conjoint analysis is widely used in market research to determine trade-offs consumers make when evaluating, selecting, and determining whether to continue to use products. It can provide key insights into what attributes are important to potential product users [23], and unlike focus groups, it does not require the participant to articulate or even identify the reasons for her preferences. This method dates back to the early 1970s, and various adaptations have been used successfully on an extremely wide range of topics [24], including previous research on microbicides and HIV prevention [25–29]. In 2012, Kinsler and colleagues found product effectiveness had the greatest impact on acceptability for hypothetical rectal microbicides among men who have sex with men (MSM) [26]. Among young women in California, Holt and coworkers found that multi-functionality, over-the counter availability, applicator insertion, and limited product leakage not requiring a panty liner were key product attributes [29].

Many previous microbicide prototypes focus on liquid gels, which have issues with leakage [30, 31], or solid forms like tablets, films, and rings, which may have other limitations like a longer time to activate [32] or, in the case of rings, the need for manual removal following use. Previous conjoint studies on microbicides were designed to understand these liquid and solid forms. Recently, we have designed a novel dosage form, specifically semi-soft suppositories made from carrageenan, that may overcome some of the issues women encountered previously with other microbicides. Falling within the intermediate design space between solids and liquids, our semi-soft suppositories are designed to both release drug relatively quickly and to breakdown in the vagina with minimal mess and leakage. As part of a broader research program on vaginal drug delivery systems [12, 13, 33–36], we are attempting to incorporate acceptability data early in the microbicide design process to iteratively arrive at an optimized vaginal delivery system. Previously, we have investigated how suppository features such as size, shape and firmness influence women’s willingness-to-try the product using an *in mano* handling protocol [12, 13, 33] with physical prototypes. We have also collected qualitative data on use preferences such as wait-time before insertion, frequency of usage, and partner awareness and acceptability in focus groups with women [37]. Simultaneously, we have quantified how different physical parameters such as firmness, size, and composition affect drug release in vitro, by measuring release of the antiretroviral drug tenofovir into Vaginal Simulant Fluid [34]. To confirm the findings of our qualitative work with focus groups in a quantitative method, the current study was designed to provide feedback for our iterative suppository design process. By building on prior efforts to understand what women functional and sensory attributes women find to be most important, we hope to formulate an ‘ideal’ soft-gel product. The present study differs from prior conjoint studies on microbicides [25–28] in two distinct ways. First, by using semi-soft gels, we are working in a novel design space that is different from prior solid or liquid product forms (most ‘gels’ that have been studied previously are not in fact gels rheologically; rather, they are thickened liquids). Second, in prior microbicide studies, images of product prototypes have not been used. Elsewhere, inclusion of product photographs have been found to be beneficial [38], so we included images of prototypes in the study described here.

Here, we obtained input from sexually active women (i.e., potential users) on the importance of various features that potentially relate to the decision to try vaginal microbicides, and we did so using a technique (i.e., conjoint analysis) that requires individuals to make implicit tradeoffs between potentially competing aspects. By decomposing the relative importance of these aspects via regression, it becomes possible to identify which aspects appeal to the user, even if they cannot articulate the reasons for their preferences. The information gained can be used to inform development of optimized forms of vaginal microbicides, and to do so pre-clinically, before such products are advanced to clinical trials that are needed to ultimately determine usage adherence and efficacy.

## Materials and Methods

### Overview

Participants completed a web based conjoint survey using the IdeaMap platform (Mind Genomics Associates, Inc. White Plains, NY). Participants were primarily recruited by flyers and word of mouth from The Pennsylvania State University campus in University Park, PA and surrounding community in State College, PA, although the online nature of the study did not preclude individuals from other locales from participating. Eligible participants were women aged 18–35 years who reported having engaged in vaginal sex with a man in the past 12 months.

### Ethics Statement

All procedures, including the consent process, were approved by the Pennsylvania State University Institutional Review Board (protocol #44741).

Participation was strictly voluntary and all responses were anonymous. Given the potentially sensitive nature of the study, written consent was not obtained to help protect participants’ anonymity. To ensure anonymity, a three-step data collection process was utilized. In the first step, participants were surveyed to ensure that they fell within the target demographic. Participants accessed this pre-screening survey through a link (URL) contained in the recruitment materials. If an individual qualified, she was given the password and redirected to the main survey (the conjoint study) hosted at the IdeaMap site. Following evaluation of product concepts, participants were asked to complete a short demographic questionnaire. At the conclusion of conjoint and demographic data collection on the IdeaMap site, participants were given another optional link which redirected them to a separate survey (powered by Survey Monkey) on a new website, where they were invited to enter a drawing for the participation incentive (an Apple iPad). This step ensured names entered in incentive drawing were completely unlinked to individual responses on the conjoint and demographic portions of the study. No attempt was made to verify the identity or demographics of study completers, as the study was anonymous by design.

### Design and Data collection

IdeaMap, is an internet-based research tool that has the capability of reaching a large number of people at a relatively low cost. The IdeaMap platform provides a modified conjoint method that can assess the effects of independent variables on concept appeal [39]. This software has been used previously in numerous studies on diverse subjects [40–44]. Briefly, in this method, product attributes to be assessed are grouped into categories and each category is composed of multiple elements. Elements from different categories are then permuted via an factorial experimental design to create unique concept vignettes. The design ensures that ratings for each of the elements will be statistically independent of each other [45]. Each participant receives a different subset of all of the possible concept vignettes and provides an overall rating for each new vignette on a 9-point scale. Critically, acceptability or importance of the individual elements are never assessed directly. The overall ratings are then decomposed via ordinary least squares regression to understand the relative appeal of each individual element. Up to four interest values (the number or elements present in each vignette) can be added to the baseline value to get an estimate of the percentage of women who would respond favorably to a given microbicide concept, where favorable is defined as a rating of 7 or higher on a 9 point scale.

**Fig 1:**
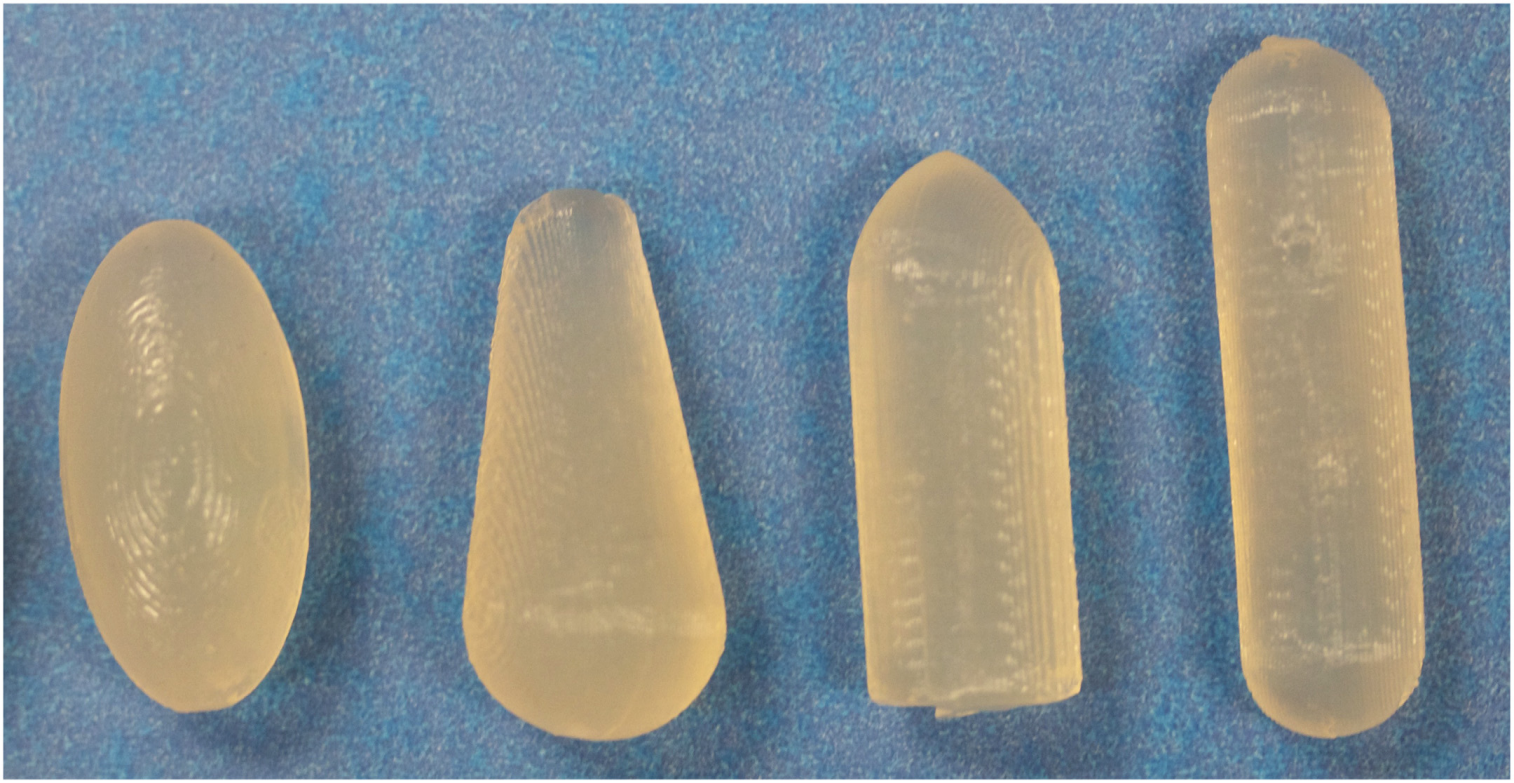
Composite image of the four shapes included in the study. Participants only ever saw one prototype photograph at a time, and not all vignettes included a photograph. From left to right, the shapes are oval, teardrop, bullet, and tampon. Participants never saw these verbal descriptors, and they are used here only for convenience.

Here, the attributes used were divided into seven categories: 1) product images; 2) messiness and leakage; 3) wait time; 4) product function; 5) partner awareness; 6) duration of protection; and 7) color. Each category was constructed of four elements, or word phrases, relating to that category. Thus, a total of 28 independent elements were included (see Table 1). Examples of elements included, but are not limited to: “the product will protect against HIV and other STDs…” “the product is translucent/clear in color,” and “the product produces some discharge, which is similar to increased vaginal discharge during sex.” Unlike prior microbicide conjoint studies [25–28], we also presented photographs of prototype microbicides to illustrate a series of potential shapes. These shapes were chosen based on previous laboratory based studies where physical prototypes were handled [12], and these are shown in Fig 1.

**Table 1:** Categories and individual elements used in study design.

**Category 1: Images/Shape**
Bullet
Tampon
Tear Drop
Oval
**Category 2: Messiness and Leakage**
The product produces a lot of discharge and will require a panty liner…
The product produces some discharge, but does not require a panty liner…
The product produces some discharge, which is similar to increased vaginal discharge during sex…
The product does not produce any discharge…
**Category 3: Wait time**
To be effective, the product will need to be inserted at least 2 minutes before sex…
To be effective, the product will need to be inserted 15 minutes before sex…
To be effective, the product will need to be inserted 30 minutes before sex…
To be effective, the product must be inserted an hour before sex…
**Category 4: Product Function**
The product will provide protection against HIV and other STDs…
The product will provide protection against HIV only…
The product can be used to prevent pregnancy…
The product will provide HIV and STD protection and can also be used to prevent pregnancy…
**Category 5: Partner Awareness**
The product will not be noticed by your sexual partner…
The product may or may not be noticed by your sexual partner…
The product will leave residue on your sexual partner…
The product will be noticed by your sexual partner…
**Category 6: Duration of Protection**
After the necessary wait time, the product will continue to work for 1 hour…
After the necessary wait time, the product will continue to work for 4 hours…
After the necessary wait time, the product will continue to work for 1 day…
After the necessary wait time, the product will continue to work for 2–3 days…
**Category 7: Color**
The product will be translucent/clear in color…
The product will be pearlescent in color (i.e. the color and shine of a pearl) in color…
The product will be white/chalky in color…
The product will be a pale, bubblegum pink in color…

**Fig 2:**
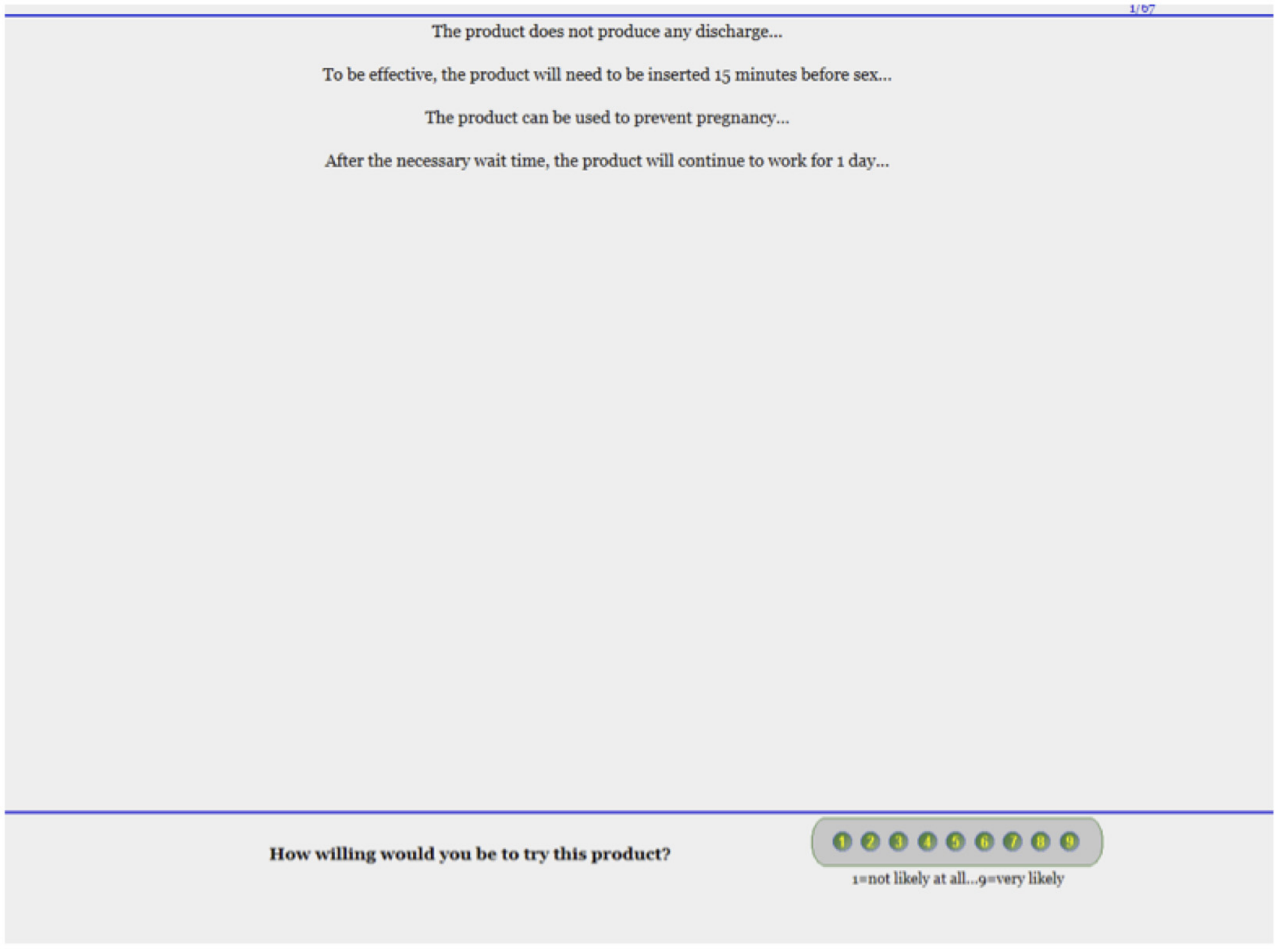
Screen shot of an example of a vignette delivered to women in the study. The various elements from Table 1 were presented in an incomplete factorial design via IdeaMap, and women rated their willingness-to-try on a 9 point scale. Each participant rated 49 vignettes, some of which included photographs of the different shapes. Using the ratings for all 302 participants across all 49 vignettes, ordinary least squares regression was then used to decompose the independent contributions of each element.

Four elements, each from a different category, were combined to create each concept vignette. For each, participants rated their willingness-to-try on a 9-point scale anchored on the left with 1 (very unlikely) and on the right with 9 (very likely). Fig 2 is a screenshot from the online survey showing one potential combination of elements (a single product concept vignette) along with the scale that was used by participants. After rating a unique set of forty-nine different vignettes generated with a incomplete factorial design, participants answered a series of demographic questions. These included questions on gender, age, race and ethnicity, employment, education level, marital status, sexual practices, STD testing and history, vaginal deliveries, and vaginal product use (Table 2). The entire survey took participants between 30 and 45 minutes to complete, inclusive of the screening, all vignettes, and the demographic questions. All project data are available for download as supporting information (S1 File).

**Table 2:** Demographic characteristics for conjoint survey participants.

Demographic Variable	n	%
**What is your gender?**		
Male	0	0%
Female	302	100%
**To which age group do you belong?**		
18–24	219	72.50%
25–29	48	15.90%
30–35	35	11.60%
36+	0	0%
Please select one or more racial categories that best describes you*.		
Caucasian or White	276	91.40%
Black or African American	7	2.30%
African	1	0.30%
American Indian or Alaskan Native	3	1.00%
Asian	6	2%
South Asian-includes India, Pakistan, Bangladesh, Nepal, Bhutan, Sri Lanka	3	1%
Native Hawaiian or other Pacific Islander	0	0%
Other	8	2.60%
Decline to Answer	4	1.30%
**Please select your select your ethnicity**		
Hispanic or Latina	14	4.60%
Not Hispanic or Latina	283	93.70%
Decline to Answer	5	1.70%
**What is your employment status?**		
Employed	68	23%
Student	227	75%
Unemployed	1	0%
Homemaker	6	2%
Retired	0	0%
**What is the highest level of education you have completed?**		
Less than High School	0	0%
High School/GED	25	8%
1 or more years of college, no degree	144	48%
Bachelor's degree (For example: BA, AB, BS)	93	31%
Master's degree (For example: MA, MS, Med, MEng, MBA)	37	12%
Professional degree (For example: MD, DDS, DVM, LLB, JD)	1	0%
Doctoral Degree (For example: PhD, EdD)	2	1%
**What is your current marital status?**		
Married	51	17%
Widowed	0	0%
Divorced	2	1%
Separated	0	0%
Single, never married	249	82%
**On average, how often have you had vaginal sex in the past 12 months?**		
Less than once per month	60	20%
2–4 times per month	114	38%
2–4 times per week	103	34%
More than 4 times per week	20	7%
Decline to answer	5	2%
**How many different male partners have you had vaginal sex with in the past 12 months?**		
One	195	65%
5-Feb	93	31%
10-Jun	9	3%
More than 10	2	1%
Decline to answer	3	1%
**What types of sex do you typically engage in (select all that apply)?**		
Vaginal	294	97%
Anal	16	5%
Oral	229	76%
Decline to answer	6	2%
**Do you typically use a lubricant during vaginal sex?**		
Yes, all the time	16	5%
Yes, occasionally	58	19%
Yes, I have tried it	99	33%
No, I have never tried one	126	42%
Decline to answer	3	1%
**Do you require your partner to wear a condom during vaginal sex?**		
Yes, all the time	114	38%
Yes, only with someone new	23	8%
Yes, occasionally	43	14%
No, we use other methods of birth control	104	34%
No, we use other methods to prevent STD transmission	1	0%
No	11	4%
Decline to answer	6	2%
**How often are you screened for STDs/HIV?**		
Annually	121	40%
Once every 2–3 years	42	14%
Every time I change my sexual partner	39	13%
Never	97	32%
Decline to answer	3	1%
**Have you ever been diagnosed with a sexually transmitted disease (STD)?**		
Yes	25	8%
No	273	90%
Decline to answer	4	1%
**Please indicate the number of vaginal deliveries you have had (do not include Cesarean Section/ “C-Section” deliveries)**		
None	282	93%
One	12	4%
Two	7	2%
Three	1	0%
Four or more	0	0%
**How often have you used a vaginal medication for yeast infections or bacterial vaginosis?**		
Frequently	3	1%
Occasionally	29	10%
Once or twice	125	41%
Never used one	145	48%
**Do you use a tampon?**		
Yes, for every cycle	234	77%
Yes, a few times a year	31	10%
Yes, I have tried it	22	7%
No, I have tried one	15	5%
**Have you ever tried a spermicidal cream/gel for birth control?**		
Yes	12	4%
No	290	96%
Decline to answer	0	0%

* category sum exceeds 100% because individuals were allowed to check more than one category; six individuals did so, 5 of whom selected ‘causasian or white’ along with another category.

### Data analysis

Analysis was performed using the integrated tools within the IdeaMap platform; these were used to determine the baseline and additive effects of the individual elements on the group’s willingness-to-try the product. During analysis, the software creates a new binary variable called ‘interest’. An interest value of one is assigned if a participant rated a vignette (i.e., product concept) 7 or higher on the willingness-to-try scale, while an interest value of zero is assigned if a participant rated the vignette below 7 on the scale. Using ordinary least squares regression, a model is created that relates the presence or absence of individual elements to the resulting binary coded interest values. A regression constant (or baseline value) is calculated as the baseline interest in the absence of any elements, and individual interest values are calculated for each element. The resulting interest values modify the baseline value, and give a relative measure of influence that an individual element has compared to the other elements [39]. A positive interest value indicates that element increases the group’s willingness-to-try for a microbicide, while a negative interest value indicates that element decreases the group’s willingness-to-try. An interest value near zero indicates the element does not meaningfully impact willingness-to-try the product. A more thorough description of the method and resulting data analysis can be found in [45].

## Results

A total of 302 sexually active women between the ages of 18 and 35 years completed the study, where sexually active was defined as having had vaginal sex with a man in the last 12 months. A summary of participant demographics is given in Table 2. The majority of participants in this study were between the ages of 18 and 24 years, self identified as White or Caucasian, and were currently university students. In addition, 65% reported only one sexual partner within the past 12 months, and 31% reported 2 to 5 partners in the past 12 months; more than half had been screened for STDs/HIV within the last 3 years. Only 8% indicated they had previously been diagnosed with a ‘sexually transmitted disease (STD)’. Accordingly, the present cohort may not be reflective of individual at high risk for HIV transmission.

**Fig 3:**
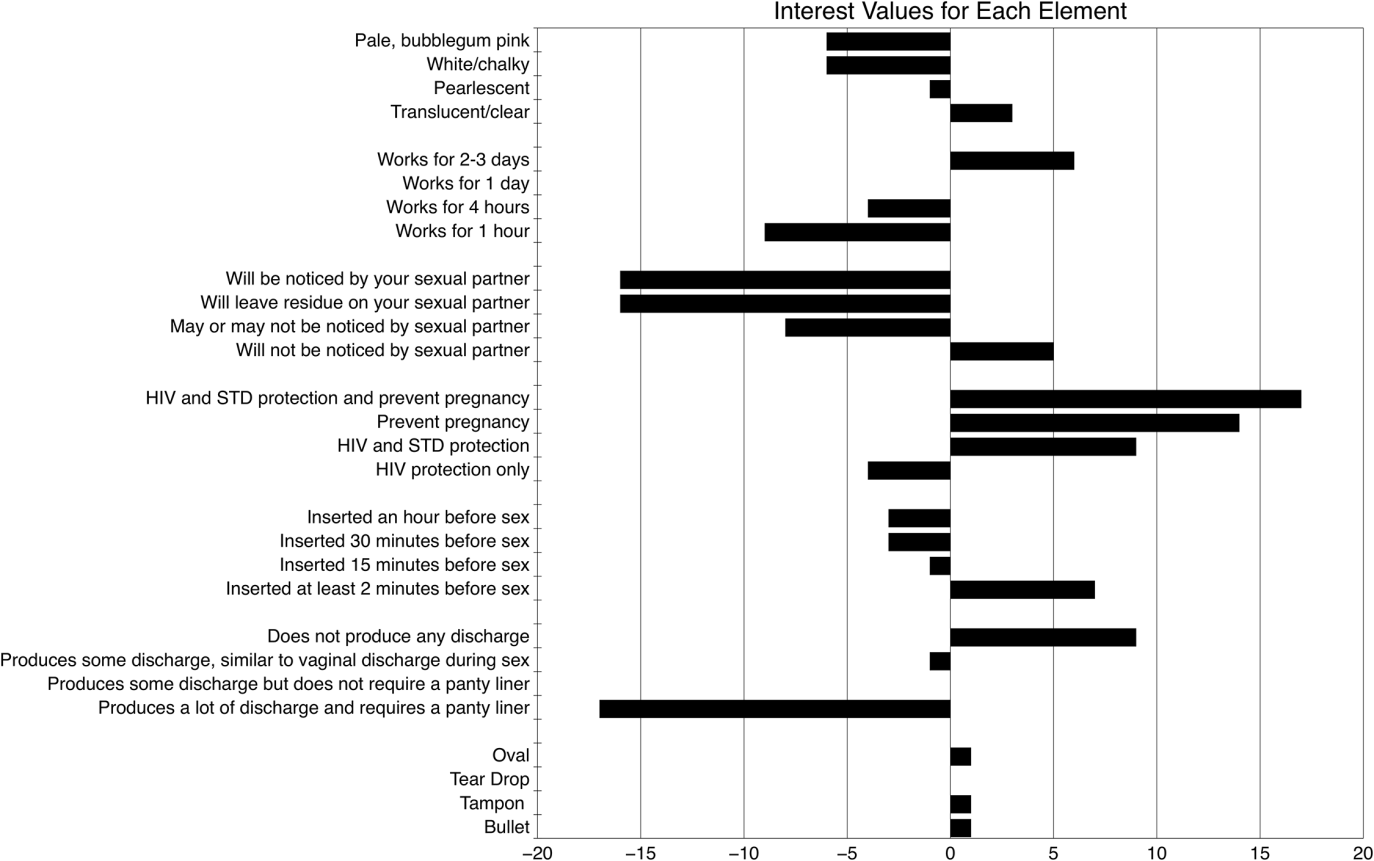
Individual interest values for all 28 elements are shown here. A larger, positive interest value indicates that element had a larger, positive impact on women’s willingness-to-try ratings. A larger, negative interest value indicates that element had a larger, negative impact on women’s willingness-to-try ratings. Up to four interest values (the number or elements present in each vignette) can be added to the baseline value to get measure of the percentage of women who would respond favorably (rating of 7 or higher in willingness-to-try) to a given microbicide concept. For example, a vaginal microbicide with multi-functionality to prevent HIV, STDs, and pregnancy (+17) that could be inserted just two minutes prior to coitus (+7) and worked for 2–3 days (+6) without producing any discharge (+9) is predicted to receive favorable ratings by 64% of the women surveyed, where favorable is defined as a score of 7 or higher on a 9 point scale. Conversely, a microbicide that works for 4 hours (-4) and produces enough discharge to require a panty liner (-17) would only be predicted to be rated at 7 or above (i.e., favorable) by 4% of women. In practice, a vaginal microbicide could be characterized by more than four positive elements, so favorability ratings could increase beyond this, but limitations of the method prevent modeling this.

The interest values of the various elements are shown in Fig 3. Eleven elements had positive interest values. Positive effects on the group’s willingness-to-try were seen with all elements related to multi-functionality (HIV, STD, and/or pregnancy protection), a short wait time for efficacy (2 minutes), a longer duration of protection (2–3 days), and the potential for covert use (low partner awareness). Two of the highest elements were from the product function category, “The product will provide HIV and STD protection and can also be used to prevent pregnancy…” and “the product can be used to prevent pregnancy…”, receiving interest values of 17 and 14, respectively.

Fourteen elements received negative interest values. All statements that suggested a long wait time before efficacy (15 minutes or more), a short duration of protection (4 hours or less), a color other than clear/translucent, and elements related to higher partner awareness all negatively influenced the group’s willingness-to-try a hypothetical vaginal microbicide. Of the three lowest scoring elements, two were related to partner awareness (“the product will leave residue on your sexual partner…” and “the product will be noticed by your sexual partner…”), each receiving interest values of -16. The 3^rd^ of lowest scoring elements was “the product produces a lot of discharge and requires a panty liner…”, which received an interest value of -17. As expected, a product that produced no discharge was appealing to women with an interest value of +9; notably however, women were willing to tolerate some discharge, as the two elements that indicated presence of ‘some discharge’ had interest values near zero. Surprisingly, when judged via photographs (versus physical evaluation in the hand, as we have done previously), product shape also had minimal effect on willingness-to-try: the tear drop shape elicited an interest value of zero, while the other three shapes elicited interest values of +1.

## Discussion

Within our participants, there were several notable trends relating to women’s preferences for a hypothetical microbicide product. Three categories, messiness and leakage, partner awareness, and product function produced a wide range of interest values, depending on the individual elements, indicating these features are highly salient to women when deciding whether to try a vaginal microbicide. Duration of protection, wait time, and product color showed moderate differences between the individual elements within a category, so these features should also be taken into account when designing vaginal microbicides. In comparison to the aforementioned factors, product shape (as assessed via photographs) appeared to be less important to women. With the exception of the shape data, these findings were generally consistent with two prior conjoint studies on microbicides. In 321 women aged 18–32 years from Northern California, Holt and colleagues [29] observed that multi-functionality and only slight leakage not requiring a panty liner were positive attributes in their conjoint survey. Among 402 mid-western urban adolescent women, important attributes included a microbicide with short application time post-coitus or pre-coitus and a microbicide with contraceptive properties [46]. Other research looking at both sensory and non-sensory microbicide attributes also show similar findings, as multi-functionality and covert use (which is related to low partner awareness) have been identified as important attributes for vaginal microbicide acceptance [47, 48]. Among men who have sex with men (MSM), conjoint analysis of rectal microbicide acceptability suggests frequency of use has a significant impact on product appeal, with higher ratings for a pre-coital regimen compared to a regimen requiring daily use regardless of sexual activity [26]; that is, coitally-associated products are preferable to coitally-independent products, at least in MSM. Formulations with a longer duration of protection also scored well, although we should note that in our study, we did not explicitly distinguish between “long acting” and “long-residence” products. Focus groups suggest these concepts are not synonymous [49], suggesting it may be worth disambiguating these two concepts in future conjoint studies. In long acting vaginal microbicide products, the vehicle erodes and comes out as vaginal discharge while the active pharmaceutical ingredient (API) enters the target tissue and continues to provide protection. Conversely, in long residence products, the vehicle persists and releases the API throughout the duration of protection. In case of the delivery form being investigated in this study, there may be some unknown residence time prior to vehicle degradation, and this needs to be investigated further. During focus groups conducted by our team, women anticipated that they, and perhaps their partner, might feel the larger and firmer ovules during coitus (Zaveri et al, submitted elsewhere). Thus, vehicle residence time needs to be evaluated in conjunction with, and in contrast to the duration of protection. Based on conjoint and focus group data, our current formulation efforts are focused on design of a suppository with a short wait time to efficacy following insertion that also provides continuous release and protection up to 48 hours [33, 34]. Consequently, we are investigating mixed polymer systems that include carrageenan create a suppository that mimics natural vaginal mucous following suppository breakdown in the vagina to facilitate low user and partner awareness.

In our participants, a single multifunctional product that prevented pregnancy and a range of STIs was highly appealing, and products focused solely on contraception, or broad spectrum STI protection also scored well. Conversely, a product that only protected against HIV was not desirable. This finding should be interpreted cautiously, both in light of our population (primarily college aged women with low numbers of male partners), and in consideration of other research showing women prefer coitally-associated usage regimens [46]. More critically, low adherence rates were observed in the VOICE trial in Africa, which failed to find any protective effect from vaginally delivered tenofovir [19]; most participants in that trial failed to use the product daily, but whether that was due to the delivery form (a messy ‘gel’) or other factors that precluded daily use is unclear. Depending on both the composition of the vehicle and the active ingredient, a vaginal suppository that is also capable of preventing pregnancy may need to be used regularly, and possibly daily, to be effective. In our prior focus groups, some women voiced preferences for spermicidal contraceptives, as compared to hormonal methods, which could be incorporated in microbicide dosage forms [37]. On the other hand, it is possible protocol adherence rates might potentially improve if product usage was linked to a practice women already perform daily (i.e. using medication daily as a contraceptive). Additional work is needed to determine how attributes that are desirable in a microbicide may or may not generalize across different populations of women in different social, political, medical and risk contexts.

In previous studies from our laboratory, physical prototypes of various geometries were evaluated *in mano* and women rated their willingness-to-try these samples [12, 33]. In one study, we found oval shaped and bullet shaped prototypes scored highest compared to round oval, teardrop, and tampon shaped prototypes [12]. Here, we found only a slight additive effect with bullet shaped, oval shaped, and tampon shaped prototypes, while the teardrop shaped prototype had no effect. It appears that other elements measured in this study had a much stronger influence on women’s willingness-to-try the prototypes. Alternatively, this may reflect methodological differences between the two studies (viewing photographs on a computer screen versus handling physical prototypes *in mano*). Additional work is required to resolve this discrepancy.

According to the National Survey of Sexual Health and Behavior, the percentage of women participating in vaginal intercourse in America is highest between ages 20 and 39: for women aged 20–24, 25–29, and 30–39 years of age, it is estimated that 80%, 87%, and 74% had engaged in vaginal intercourse in the last year [50]. While we targeted this age range to gain a better understanding of the attributes that affect a woman’s willingness-to-try hypothetical microbicide products, the generalizability of our findings may be limited by our sample: our data were collected primarily from college educated white women who were recruited from a large land grant university in central Pennsylvania. Previously, it was reported that women with higher levels of education were more willing to try a hypothetical vaginal product [51]. Also, most of these women reported only having one male partner in the past 12 months. Additional work is needed to confirm present findings hold true in other populations, especially those at elevated risk for HIV exposure. Nonetheless, present data were also largely consistent with other conjoint studies collected in other age ranges in other geographic locations.

Finally, the hypothetical nature of the present study should be acknowledged. It is well known in behavioral health research that stated intentions and preferences often fail to translate into real world choices and actions. For example, in the VOICE trial, most women failed to use the study products daily, in spite of pre-study assessments indicating otherwise [19]. Here, women were not actually required to use microbicides or microbicide surrogates vaginally. In vivo testing was not attempted both because the funding mechanism for the present work specifically precludes first in human studies and because safety data is not yet available for this delivery system. Nonetheless, we still believe important insights for microbicide development can be gained in preclinical research like the present study. Clinical trials are invasive, time consuming, and expensive, so it is more cost effective to rule out dosage forms and usage parameters that are non-starters in terms of compliance and adherence, even if they might be biologically efficacious. That is, while it is quite possibly that a microbicide with high acceptability in hypothetical ex vivo testing may still fail in a clinical trial due to factors that only become apparent with use, it is also highly unlikely that a microbicide with low willingness-to-try ex vivo can ever be successful in vivo. Thus, eliminating these forms or usage conditions very early in the development process is prudent (i.e., stage gating), and doing so by obtaining empirical input directly from potential users helps avoids situations in which the formulator must make design tradeoffs based on his or her own beliefs and assumptions about what women do or do not what. The present conjoint study is part of a larger development effort (e.g., [12, 13, 33]), and serves as a proof of concept on how insight on the relative weights of various factors that influence microbicide acceptability can be gained rapidly and efficiently using internet-based methods. Consumer driven product development is a large, mature field that uses many complimentary approaches, and these techniques can readily be adapted to microbicide development.

## Conclusions

Development of an effective microbicide product remains a challenge from both a product formulation and user acceptability standpoint. However, present data suggests microbicide developers should focus on multi-functionality, while ensuring a clear/translucent colored product that will not cause leakage or be noticed by a women’s sexual partner. These insights support our recent efforts to formulate a firmer soft-gel suppository [12, 13, 33, 34] vis-à-vis more liquid-like microbicides [52] or microbicide surrogates [35, 36]. While the information collected here is informative, additional research on the ideal attributes of potential microbicide products is warranted. Future work by our group will expand this approach to a more racially, geographically, and socioeconomically diverse population. Additional work is also needed to determine if the vaginal microbicide market is relatively homogeneous; alternatively, different market segments may potentially exist for microbicides if women of different risk or demographics hold divergent preferences regarding microbicide attributes.

## Supporting Information

S1 File Complete Datafiles. A single compressed zip file containing the entire dataset used here is provided as supporting information. This zip file includes five tab delimited datafiles and a readme txt file indicating what is found in each datafile.
(ZIP)

## References

[1] Global report: UNAIDS report on the global AIDS epidemic: 2012. UNAIDS, 2012 9291739960.

[2] Global incidence and prevalence of selected curable sexually transmitted infections—2008. World Health Organization, 2012.

[3] Prevention CoDCa. Incidence, Prevalence, and Cost of Sexually Transmitted Infections in the United States2013; 2014 (November 18, 2014). Available: http://www.cdc.gov/std/stats/sti-estimates-fact-sheet-feb-2013.pdf.

[4] HIV Among Women. Centers of Disease Control and Prevention, 2014 Contract No.: November 18, 2014.

[5] Pennsylvania—2013 State Health Profile. Centers of Disease Control and Prevention 2013 Contract No.: November 18, 2014.

[6] Fact Sheet About Microbicides. Microbicide Trials Network, 2014 Contract No.: November 18, 2014.

[7] Akil A, Parniak MA, Dezzuitti CS, Moncla BJ, Cost MR, Li M, et al Development and Characterization of a Vaginal Film Containing Dapivirine, a Non- nucleoside Reverse Transcriptase Inhibitor (NNRTI), for prevention of HIV-1 sexual transmission. Drug delivery and translational research. 2011;1(3):209–22. 10.1007/s13346-011-0022-6 22708075 PMC3375737

[8] Cole AM, Patton DL, Rohan LC, Cole AL, Cosgrove-Sweeney Y, Rogers NA, et al The Formulated Microbicide RC-101 Was Safe and Antivirally Active Following Intravaginal Application in Pigtailed Macaques. PLoS ONE. 2010;5(11):e15111 10.1371/journal.pone.0015111 21124745 PMC2993972

[9] Devlin B, Nuttall J, Wilder S, Woodsong C, Rosenberg Z. Development of dapivirine vaginal ring for HIV prevention. Antiviral Research. 2013;100, Supplement(0):S3–S8. 10.1016/j.antiviral.2013.09.025 24188702

[10] Fetherston SM, Boyd P, McCoy CF, McBride MC, Edwards K-L, Ampofo S, et al A silicone elastomer vaginal ring for HIV prevention containing two microbicides with different mechanisms of action. European Journal of Pharmaceutical Sciences. 2013;48(3):406–15. 10.1016/j.ejps.2012.12.002 23266465

[11] Hoffman S, Morrow K, Mantell J, Rosen R, Carballo-Diéguez A, Gai F. Covert Use, Vaginal Lubrication, and Sexual Pleasure: A Qualitative Study of Urban U.S. Women in a Vaginal Microbicide Clinical Trial. Arch Sex Behav. 2010;39(3):748–60. 10.1007/s10508-009-9509-3 19636696 PMC2855760

[12] Li B, Zaveri T, Ziegler GR, Hayes JE. Shape of vaginal suppositories affects willingness-to-try and preference. Antiviral Research. 2013;97(3):280–4. 10.1016/j.antiviral.2012.12.024 23276592 PMC3608716

[13] Li B, Zaveri T, Ziegler GR, Hayes JE. User Preferences in a Carrageenan-Based Vaginal Drug Delivery System. PLoS One. 2013;8(1). 10.1371/journal.pone.0054975 PMC3554666 23358688

[14] Hammett TM, Mason TH, Joanis CL, Foster SE, Harmon P, Robles RR, et al Acceptability of Formulations and Application Methods for Vaginal Microbicides Among Drug‐Involved Women: Results of Product Trials in Three Cities. Sexually Transmitted Diseases. 2000;27(2):119–26. 10676980 10.1097/00007435-200002000-00011

[15] Robertson AM, Syvertsen JL, Martinez G, Rangel MG, Palinkas LA, Stockman JK, et al Acceptability of vaginal microbicides among female sex workers and their intimate male partners in two Mexico—US border cities: A mixed methods analysis. Global Public Health. 2013;8(5):619–33. 10.1080/17441692.2012.762412 23398385 PMC3674154

[16] Grant RM, Lama JR, Anderson PL, McMahan V, Liu AY, Vargas L, et al Preexposure chemoprophylaxis for HIV prevention in men who have sex with men. New England Journal of Medicine. 2010;363(27):2587–99. 10.1056/NEJMoa1011205 21091279 PMC3079639

[17] Karim QA, Karim SSA, Frohlich JA, Grobler AC, Baxter C, Mansoor LE, et al Effectiveness and safety of tenofovir gel, an antiretroviral microbicide, for the prevention of HIV infection in women. science. 2010;329(5996):1168–74. 10.1126/science.1193748 20643915 PMC3001187

[18] Karim SSA, Karim QA. Antiretroviral prophylaxis: a defining moment for HIV prevention. Lancet. 2011;378(9809):e23 10.1016/S0140-6736(11)61136-7 21771566 PMC3253379

[19] Marrazzo JM, Ramjee G, Richardson BA, Gomez K, Mgodi N, Nair G, et al Tenofovir-Based Preexposure Prophylaxis for HIV Infection among African Women. New England Journal of Medicine. 2015;372(6):509–18. 10.1056/NEJMoa1402269 . 25651245 PMC4341965

[20] Rees H, Delaney-More S, Baron D, Lombard C, Gray G, Myer L, et al. editor FACTS 001 Phase III Trial of Pericoital Tenofovir 1% Gel for HIV Prevention in Women. Conference on Retroviruses and Opportunistic Infections (CROI); 2015; Seattle, WA.

[21] van der Straten A, Stadler J, Montgomery E, Hartmann M, Magazi B, Mathebula F, et al Women’s Experiences with Oral and Vaginal Pre-Exposure Prophylaxis: The VOICE-C Qualitative Study in Johannesburg, South Africa. PLoS ONE. 2014;9(2):e89118 10.1371/journal.pone.0089118 24586534 PMC3931679

[22] Rosen M, van den Berg JJ, Vargas S, Senocak N, Shaw JG, Buckheit RW, et al Meaning-making matters in product design: Users’ sensory perceptions and experience evaluations of long-acting vaginal gels and intravaginal rings. Contraception. 2015. Epub 11th August 2015. 10.1016/j.contraception.2015.08.007 PMC4664151 26276246

[23] Green PE, Krieger AM, Wind Y. Thirty years of conjoint analysis: Reflections and prospects. Interfaces. 2001;31(3_supplement):S56–S73.

[24] Green PE, Srinivasan V. Conjoint analysis in marketing: new developments with implications for research and practice. The Journal of Marketing. 1990:3–19.

[25] Beusterien KM, Dziekan K, Flood E, Harding G, Jordan JC. Understanding Patient Preferences for HIV Medications Using Adaptive Conjoint Analysis: Feasibility Assessment. Value in Health. 2005;8(4):453–61. 10.1111/j.1524-4733.2005.00036.x 16091022

[26] Kinsler JJ, Cunningham WE, Nureña CR, Nadjat-haiem C, Grinsztejn B, Casapia M, et al Using Conjoint Analysis to Measure the Acceptability of Rectal Microbicides Among Men Who Have Sex with Men in Four South American Cities. AIDS and Behavior. 2012;16(6):1436–47. 10.1007/s10461-011-0045-5 1027176583; . 21959986

[27] Lee SJ, Newman PA, Comulada WS, Cunningham WE, Duan N. Use of conjoint analysis to assess HIV vaccine acceptability: feasibility of an innovation in the assessment of consumer health-care preferences. International journal of STD & AIDS. 2012;23(4):235–41. 10.1258/ijsa.2011.011189 22581945 PMC3372064

[28] Weaver JMPH, Newman PAP, Williams CCP, Massaquoi NMSW, Brown MMSW. "Sisters, Mothers, Daughters and Aunties": HIV Vaccine Acceptability Among African, Caribbean and Other Black Women in Toronto. Canadian Journal of Public Health. 2013;104(5):e413–7. 1459652402; . 24183184 10.17269/cjph.104.3915 PMC6974058

[29] Holt BY, Morwitz VG, Ngo L, Harrison PF, Whaley KJ, Pettifor A, et al Microbicide preference among young women in California. Journal of Women's Health. 2006;15(3):281–94. 16620187 10.1089/jwh.2006.15.281

[30] Giguere R, Carballo-Dieguez A, Ventuneac A, Mabragana M, Dolezal C, Chen BA, et al Variations in microbicide gel acceptability among young women in the USA and Puerto Rico. Cult Health Sex. 2012;14(2):151–66. 10.1080/13691058.2011.630099 ISI:000301710100003. 22084840 PMC3265079

[31] Morrow K, Rosen R, Richter L, Emans A, Forbes A, Day J, et al The acceptability of an investigational vaginal microbicide, PRO 2000 gel, among women in a phase I clinical trial. Journal of Women's Health. 2003;12(7):655–66. 10.1089/154099903322404302 ISI:000188439100005. 14583106

[32] Nel AM, Mitchnick LB, Risha P, Muungo LT, Norick PM. Acceptability of vaginal film, soft-gel capsule, and tablet as potential microbicide delivery methods among African women. Journal of Women's Health (Larchmt). 2011;20(8):1207–14. Epub 2011/07/22. 10.1089/jwh.2010.2476 . 21774672

[33] Zaveri T, Primrose RJ, Surapaneni L, Ziegler GR, Hayes JE. Firmness Perception Influences Women's Preferences for Vaginal Suppositories. Pharmaceutics. 2014;6(3):512–29. 10.3390/pharmaceutics6030512 25211123 PMC4190533

[34] Zaveri T, Hayes JE, Ziegler GR. Release of tenofovir from carrageenan-based vaginal suppositories. Pharmaceutics. 2014;6(3):366–77. 10.3390/pharmaceutics6030366 24999606 PMC4190525

[35] Mahan ED, Zaveri T, Ziegler GR, Hayes JE. Relationships between perceptual attributes and rheology in over-the-counter vaginal products: a potential tool for microbicide development. PLoS One. 2014;9(9):e105614 Epub 2014/09/05. 10.1371/journal.pone.0105614 25188244 PMC4154878

[36] Mahan ED, Morrow KM, Hayes JE. Quantitative perceptual differences among over-the-counter vaginal products using a standardized methodology: implications for microbicide development. Contraception. 2011;84(2):184–93. Epub 2011/07/16. S0010-7824(10)00658-X [pii] 10.1016/j.contraception.2010.11.012 . 21757061 PMC4699565

[37] Zaveri T, Powell KA, Li B, Ziegler GR, Hayes JE, editors. Improving acceptability of vaginal drug delivery systems by using sensory methods. The Society of Sensory Professionals 3rd Technical and Professional Conference; 2012 Oct 10–12; Jersey City, New Jersey.

[38] Jansen S, Boumeester H, Coolen H, Goetgeluk R, Molin E. The impact of including images in a conjoint measurement task: evidence from two small-scale studies. J Hous and the Built Environ. 2009;24(3):271–97. 10.1007/s10901-009-9149-x

[39] MJI-DesignLab. IdeaMap(R) White Plains, NY 2014 [20 August 2014.].

[40] Foley M, Beckley J, Ashman H, Moskowitz HR. The mind-set of teens towards food communications revealed by conjoint measurement and multi-food databases. Appetite. 2009;52(3):554–60. 10.1016/j.appet.2009.01.006 19501750

[41] Moskowitz HR, German JB, Saguy IS. Unveiling health attitudes and creating good-for-you foods: the genomics metaphor, consumer innovative web-based technologies. Critical reviews in food science and nutrition. 2005;45(3):165–91. 16048147 10.1080/10408690590956350

[42] Saulo AA, Moskowitz HR. Uncovering the mind-sets of consumers towards food safety messages. Food quality and preference. 2011;22(5):422–32.

[43] Levin LA, Langer KM, Clark DG, Colquhoun TA, Callaway JL, Moskowitz HR. Using Mind Genomics to Identify Essential Elements of a Flower Product. HortScience. 2012;47(11):1658–65.

[44] Colquhoun TA, Levin LA, Moskowitz HR, Whitaker VM, Clark DG, Folta KM. Framing the perfect strawberry: An exercise in consumer-assisted selection of fruit crops. Journal of Berry Research. 2012;2(1):45–61.

[45] Moskowitz HR. 'Mind genomics': the experimental, inductive science of the ordinary, and its application to aspects of food and feeding. Physiol Behav. 2012;107(4):606–13. . 22542473 10.1016/j.physbeh.2012.04.009

[46] Tanner AE, Katzenstein JM, Zimet GD, Cox DS, Cox AD, Fortenberry JD. Vaginal Microbicide Preferences Among Midwestern Urban Adolescent Women. The Journal of adolescent health: official publication of the Society for Adolescent Medicine. 2008;43(4):349–56. 10.1016/j.jadohealth.2008.02.017 18809132 PMC2593640

[47] Tanner AE, Zimet G, Fortenberry JD, Reece M, Graham C, Murray M. Young Women's Use of a Vaginal Microbicide Surrogate: The Role of Individual and Contextual Factors in Acceptability and Sexual Pleasure. The Journal of Sex Research. 2009;46(1):15–23. 10.1080/00224490802398407 19012060

[48] Terris-Prestholt F, Hanson K, MacPhail C, Vickerman P, Rees H, Watts C. How Much Demand for New HIV Prevention Technologies Can We Really Expect? Results from a Discrete Choice Experiment in South Africa. PLoS One. 2013;8(12):e83193 10.1371/journal.pone.0083193 24386160 PMC3875434

[49] van den Berg JJ, Rosen RK, Bregman DE, Thompson LA, Jensen KM, Kiser PF, et al "Set it and forget it": women's perceptions and opinions of long-acting topical vaginal gels. AIDS Behav. 2014;18(5):862–70. Epub 2013/11/20. 10.1007/s10461-013-0652-4 24248674 PMC4018755

[50] National Survey of Sexual Health and Behavior. Center for Sexual Health Promotion, 2010 Contract No.: November 18, 2014.

[51] Reiff M, Wade C, Chao MT, Kronenberg F, Cushman LF. Health Practices and Vaginal Microbicide Acceptability among Urban Black Women. Journal of Women's Health. 2008;17(8):1345–51. 10.1089/jwh.2008.0886 18788991 PMC2944437

[52] Morrow KM, Rosen R, Richter L, Emans A, Forbes A, Day J, et al The acceptability of an investigational vaginal microbicide, PRO 2000 Gel, among women in a phase I clinical trial. J Womens Health (Larchmt). 2003;12(7):655–66. Epub 2003/10/30. 10.1089/154099903322404302 . 14583106

